# Knowledge of sickle cell disease and associated factors among undergraduate students at Makerere University: a cross-sectional study

**DOI:** 10.4314/ahs.v25i3.15

**Published:** 2025-09

**Authors:** Amon Kanyesigye, Philliam Jabim, Clinton Ariho, Nkahebwa Asasira, Isaac Kigozi, Jimmy Asiimwe Tumwine, Juliet Natukunda, Sarah Kiguli, Lydia Nabawanuka Namakula, Rawlance Ndejjo

**Affiliations:** 1 Department of Disease Control and Environmental Health, School of Public Health, Makerere University, Kampala, Uganda; 2 Department of Pharmacy, College of Health Sciences, Makerere University, Kampala, Uganda; 3 Department of Medicine, School of Medicine, College of Health Sciences, Makerere University, Kampala, Uganda; 4 Department of Nursing, School of Health Sciences, College of Health Sciences, Makerere University, Kampala, Uganda; 5 Department of Pediatrics and Child Health, College of Health Sciences, Makerere University, Kampala, Uganda

**Keywords:** Knowledge, sickle cell, students, Uganda

## Abstract

**Background:**

Sickle Cell Disease (SCD) is a hereditary disorder with significant public health implications such as chronic pain, and premature mortality. The disease is majorly in sub-Saharan Africa including Uganda. Amidst all this, research has reported low levels of awareness especially among young people such as university students who are the next generation of parents hence the need to conduct the research.

**Objectives:**

This study determined the knowledge of Sickle Cell Disease and its associated factors among undergraduate students at Makerere University.

**Methods:**

This was only a quantitative cross-sectional study conducted across 10 Colleges at Makerere University among three hundred eighty-five (385) undergraduate students. The students were randomly selected and data collected using an online structured questionnaire designed using Kobo Collect application. Collected data was analysed using logistic regression in STATA 17 software to assess the association between knowledge and demographic factors.

**Results:**

Most participants were between the ages of 18 and 22 (62.08%). While most students had heard of SCD (94.81%), a significant proportion reported that they did not know where SCD testing is done (55.06%) and had never tested for Sickle Cell Trait (92.73%). The mean knowledge score was 58.40%, with 84.68% having poor knowledge. Factors significantly associated with high SCD knowledge included being of Muslim religion (p=0.027) and knowing someone who had ever had SCD (p=0.030).

**Conclusions:**

This study reveals low knowledge levels of SCD among undergraduate students at Makerere University, with higher knowledge associated with Muslim religion and knowing someone with SCD, highlighting the need for targeted educational interventions.

## Introduction

Sickle Cell Disease (SCD) is a collective term for a collection of hemoglobinopathies characterized by the existence of 2 β-globin gene mutations or deletions with at least one being a point mutation that leads to the production of haemoglobin S (Hgb S). SCD affects about 25 million people globally and is associated with significant chronic end-organ damage, hymolytic anemia, and early death^1^. SCD is the most common haematologic condition arising from the inheritance of two haemoglobin genes that are abnormal from both parents resulting in the development of sickle-shaped red blood cells, unlike the round shape of normal red blood cells. One of the risk factors for SCD is an individual being heterozygous for sickle haemoglobin or rather individuals with the Sickle Cell Trait (SCT)^2^.

Globally, around 300,000 babies are born with SCD every year with the disease mostly prevalent in sub-Saharan Africa, India, the Mediterranean, and the Middle East^3^. Moreover, around 5% of the world's population and over 7% of pregnant women are carriers of a hemoglobinopathy that at times manifests as SCD. Also, it is estimated that over 85% of new-born babies affected with SCD occur in Africa with over 70% of cases registered in Western Africa, 17% in Central Africa and 10% in East Africa^4^. Uganda has a high national prevalence of SCT at 13.3% and disease at 0.7%^5^. Regional disparities exist in the distribution of the disease ranging from 2.5% to 23.9% with the highest prevalence in East Central and Mid-Northern Regions^5^,^6^. Furthermore, over 20,000 babies are born with SCD per year and of this, 50% to 80% die before their 5th birthday^5^. The disease also significantly contributes to poor quality of life, poor education, and burdens the health system^7^.

Uganda has also been cited as a country that has one of the highest burdens of SCD. However, previous research on SCD has reported low levels of awareness of the disease among the general population^8^. Furthermore, there is an urgent concern surrounding the increasing number of young medical students whose lives were tragically cut short due to SCD. According to a report from daily monitor^9^, three young doctors – one medical intern from Uganda Christian University and two from Makerere University lost their lives to SCD within just three years. Moreover, little is known of the awareness of SCD among university students, who are a relatively well-educated group and usually on the verge of choosing lifelong partners. This study therefore assessed the knowledge of SCD and associated factors among undergraduate students at Makerere University in Uganda.

## Methods

### Study Design and Participants

This cross-sectional study was conducted among undergraduate students at Makerere University, located in Kampala, Uganda. Makerere is the largest and oldest university in the country with a student population of over 40,000. The university is organized around 10 colleges. Within the colleges, there are different schools (31 in total) and within schools, there exist various programs that the students are undertaking. The University offers both arts and science courses. The university offers programs for about 36,000 undergraduates and 4,000 postgraduates.

### Sample size and sampling

A sample size of 385 participants was determined using the Kish Leslie (1965) formula^10^ with the assumptions of: standard normal deviate at 95% confidence interval (CI) = 1.96, assumed prevalence of sickle cell of 50% to obtain the maximum value, and margin of error of 0.05.

To select study participants, Colleges with more than three school s had three schools randomly selected using systematic probability sampling where the schools within the colleges were randomly listed and the school obtained by selecting the first school on the list and skipping the next school then selecting the one after the skip and the cycle continued, until three schools were reached. For colleges with three or fewer schools, all schools were included in the study. An equal number of study participants from each school was then obtained. Within the schools, students to participate in the study were randomly picked, irrespective of their programmes of study as the sampling frame comprised all registered undergraduate students at Makerere University during the study period. Lists of enrolled students were obtained at the school levels, from which participants were randomly selected using simple random sampling. This was done till the required sample size was was reached. The study was conducted from May to August 2023.

### Data Collection

Data were collected using an online structured questionnaire, designed using Kobo Collect based on previous literature^11^. The questionnaire was then interviewer administered to the participants by trained research assistants. The questionnaire included sections on sociodemographic characteristics, awareness and information about SCD, and knowledge of SCD. Awareness of SCD was assessed by asking participants if they had ever heard of SCD. Those who answered affirmatively were further asked about the sources of their information and whether they knew where SCD testing is done. Participants were also asked if they had ever tested for SCT and if they knew their own SCT status. Knowledge of SCD was assessed through a series of 15 questions with domains to its causes of SCD, symptoms of SCD, inheritance patterns, prevention and management strategies. Sociodemographic variables such as age, sex, marital status, college of study, year of study, and residence were also recorded.

### Data management and analysis

Following data collection, data were downloaded and imported into STATA 17 statistical software for cleaning and analysis. Descriptive statistics were used to summarize the socio-demographic characteristics of the participants, awareness, and knowledge levels. Using 15 questions, each correct response was assigned a score of 1 point, and incorrect responses were assigned a score of 0 points. With the mean knowledge score in our study being 58.40%, with the majority of participants scoring below 80%, this supported the use of binary categorization to clearly differentiate between poor and high knowledge levels. The knowledge score was then categorized into “low knowledge” (0-79%) and “high knowledge” (80-100). Logistic regression was used to examine associations between knowledge levels and socio-demographic variables at bivariate and multivariate levels. A p-value of <0.05 was considered statistically significant.

### Ethical Considerations

This study received ethical approval from the Research and Ethics Committee of Makerere University School of Public Health no. SPH-2023-374, and registered with the UNCST HS2823ES and written informed consent obtained. Prior to data collection, all participants were first briefed about the study's objectives, risks, and benefits then provided written informed consent before participating in the study. They provided written consent by signing a printed hard copy consent form before proceeding with the survey and their privacy and confidentiality were ensured.

## Results

### Sociodemographic and University characteristics of the participants

A total of 385 participants took part in the study with 202 (52.47%) females and 183 (47.53%) males. The mean age of the participants was 22.53 (standard deviation ± 2.44 years) with most 239 (62.08%) within the age range of 18 – 22 years. Almost all the participants 362 (94.03%) were single, and majority were Christians 341 (88.57%). Majority of the participants were from colleges offering science-based courses 232 (60.26, 95% CI), more than half were in year one and two (56.36%) and majority resided in hostels around the University 141 (36.62%). Details are presented in Table 1.

**Table 1:** Sociodemographic and university characteristics of the participants

Variable	Frequency	Percentage
	N=385	100 %
Age (Years)		
18-22	239	62.08
23-40	146	37.92
Sex		
Male	183	47.53
Female	202	52.47
Marital Status		
Single	362	94.03
Married/dating	23	5.97
Religion		
Moslem	39	10.13
Christian	341	88.57
Non-believer	5	1.30
College of study		
Science based college	232	60.26
Art based college	153	39.74
Year of study		
Year one and two	217	56.36
Year three and above	168	43.64
Residence		
Hall	106	27.53
Hostel	141	36.62
Home / Rental housing	138	35.85

Awareness and SCD information

Most 365 (94.81%) of the participants had heard of SCD though majority 212 (55.06%) did not know where SCD testing was done and almost all participants 357 (92.73%) had never tested for SCT. Most participants 364 (94.55%) did not know their sickle cell trait and majority 321 (83.38%) had ever heard about someone with SCD. The various sources of information about SCD are also shown in Table 2 with school 269 (69.87%) the most common.

**Table 2:** Awareness and SCD information table

Variable	Frequency	Percentage
	N=385	100 %
Have you ever heard about SCD		
Yes	365	94.81
No	20	5.19
*From where did you hear about SCD		
School	269	69.87
Health Facility	79	20.52
Social media and mass media	137	35.58
Family and friends	167	43.38
Do you know where SCD testing is done		
Yes	173	44.94
No	212	55.06
Have you ever tested for Sickle Cell Trait		
Yes	28	7.27
No	357	92.73
Do you know your Sickle Cell Trait		
Yes	21	5.45
No	364	94.55
Have you ever heard of someone with SCD		
Yes	321	83.38
No	64	16.62

* Multiple Choice

### Knowledge of SCD

The mean knowledge score was 58.40±16.23 (16.27%), indicating low general knowledge of participants. Of the 385 participants, 326 (84.68%) had poor knowledge (scored 0-79%) and 59 (15.32%) had high knowledge (scored over 80%).

### Factors associated with high knowledge levels of SCD

Results from the multivariate analysis revealed that compared to Christians, Muslims had higher odds of having high knowledge of SCD (p=0.027) and that those who knew of someone who had ever had SCD also had higher knowledge compared to their counterparts (p=0.030) (Table 3).

**Table 3:** 

Variable	Knowledge on SCD n (%)	Crude Odds ratios (95% CI)	p-value	Adjusted ORs (95% CI)	p-value
Age (Years)					
18-22	30 (50.85)	1		1	
23-40	29 (49.15)	1.73 (0.99 - 3.02)	0.055	1.55 (0.80 – 2.99)	0.185

Sex					
Female	26 (44.07)	1		1	
Male	33 (55.93)	1.48 (0.85 - 2.60)	0.162	1.40 (0.75 - 2.61)	0.287

Religion					
Christians	48 (81.34)	1		1	
Muslims	11 (18.64)	2.51 (1.17 - 5.39)	**0.018**	2.45 (1.10 - 5.44)	**0.027**

Marital Status					
Single	54 (91.53)	1			
Married/dating	5 (8.47)	1.58 (0.56 - 4.45)	0.382	-	-

Residence					
Hall	20 (33.90)	1		1	
Home	11 (18.54)	0.66 (0.29 - 1.46)	0.303	0.52 (0.22 - 1.23)	0.139
Hostel	21 (35.59)	0.75 (0.38 - 1.47)	0.407	0.82 (0.39 - 1.69)	0.596
Rental	7 (11.86)	0.63 (0.25 - 1.59)	0.326	0.63 (0.24 - 1.65)	0.346

Year of study					
One	11 (18.64)	1		1	
Two	19 (32.20)	0.65 (0.29 – 1.45)	0.293	0.71 (0.30 – 1.65)	0.424
Three	19 (32.20)	0.78 (0.34 – 1.16)	0.548	0.82 (0.35 – 1.94)	0.659
Four	6 (10.17)	1.21 (0.39 – 3.67)	0.737	0.90 (0.27 – 2.96)	0.867
Five	4 (6.78)	6.18 (1.21 – 31.63)	**0.029**	3.00 (0.51 – 17.56)	0.222

Have you ever heard of someone with SCD					
No	4 (6.78)	1		1	
Yes	55 (93.22)	2.85 (0.99 - 8.19)	0.052	3.85 (1.14 – 13.02)	**0.030**

## Discussion

This study aimed to determine the knowledge of Sickle Cell Disease (SCD) and its associated factors among undergraduate students at Makerere University. The study found that almost all students had heard of SCD, however, only 59 (15.32%) had high knowledge of SCD. The factors associated with having high SCD knowledge were Muslim religion and knowing someone who had ever had SCD.

Majority of the participants reported having heard about SCD, indicating a relatively high level of awareness within the university community. However, knowledge of SCD was low. These findings are in line with previous research among the community in Uganda^12^, Nigeria^13^,^14^ and Rwanda^15^. The low knowledge of SCD among university students could be attributed to limited attention to the disease, a lack of awareness programmes, and largely poor health seeking behaviours. From the results, the primary sources of information for students were schools, friends and family and social media. This reemphasizes the importance of the university in ensuring improved awareness and knowledge of health conditions. The significance of SCT testing and the possible repercussions of SCD for affected persons and their families should further be emphasized in university health programmes. Although there is a low amount of knowledge and fair amount of awareness, continuing efforts should be made to reinforce students' understanding of SCD, particularly the most recent advancements in its prevention and management. Social media is also important and should be tapped into to share information on SCD with university students.

A small percentage 7.27% had been tested for Sickle Cell Trait, with an even smaller proportion 5.45% aware of their own status. This demonstrates the gap that currently exists between awareness and action, emphasizing the need for more counselling and education programs to help people become aware of their SCD status and, as a result, avoid future difficulties. To increase undergraduate students' understanding of SCD, Ministry of Health should think about putting in place focused educational initiatives.

Muslims demonstrated higher odds of having high knowledge of SCD when compared to Christians, indicating differences in awareness across religious groups. This is an association that requires further exploration. In previous studies, religion did not show any association with SCD knowledge^16^,^12^,^17^ except for a study^18^ which found that religious beliefs, teachings, doctrines, and religious regulations could influence healthcare utilization such as by impeding follow-up with antenatal care visits by pregnant mothers. Muslims could therefore be stricter on religious marriages hence necessitating the need for health tests such as SCD, HIV and compatibility tests to be conducted as well. This finding therefore highlights the importance of cultural and religious factors in shaping awareness and understanding of health-related issues such as SCD and that religious institutions could play a key role in driving awareness, knowledge and screening. Additionally, individuals who knew someone with SCD exhibited higher knowledge levels compared to those who did not. This might be because of the influence of personal connections and lived experiences in shaping understanding and awareness of the disease. SCD is a unique disease that confers a significant experience to those who have experienced it or known those who have had it. In several studies, knowing someone who has ever had the disease has been a significant predictor of screening^2^,^6^,^8^,^11^. This result underscores the need to have more platforms for sharing information and lived experiences as a mechanism to not only increase knowledge, but also implore screening. The university structures such as university hospital, student clubs and associations can also play a key role in this. These findings also underscore the complex interplay of socio-demographic factors and personal experiences in shaping knowledge levels regarding SCD, highlighting the need for targeted educational interventions tailored to diverse community needs and backgrounds.

Further investigations should go more deeply into the factors associated with impacting university students' knowledge of SCD, including how curricular content and instructional strategies affect students' awareness and comprehension, cultural beliefs and family history. This would offer information for creating interventions that are successful and catered to Ugandan students' needs.

### Strengths and limitations

A strength of this study is that our findings provide a powerful indicator to guide policy-making regarding SCD, and it could serve as a benchmark for other studies. Additionally, the study's focus on identifying sources of information about SCD and factors associated with awareness contributes valuable insights for designing targeted educational interventions. Use of an interviewer administered online questionnaire was also a strength as it ensured completeness of data and reduced response bias that could otherwise arise from participants referring to online sources of information during the survey hence ensuring accurate data is collected. The limitations of this study included the reliance on self-reported data which may have introduced response bias, potentially influencing the accuracy of reported awareness and knowledge levels. Additionally, the study's sample may not be fully representative of the broader population, as it primarily included individuals from specific settings such as educational institutions. Therefore, caution should be exercised when generalizing the findings to other populations or regions. Furthermore, the study's scope was limited to assessing awareness and knowledge levels, overlooking other important factors such as attitudes, beliefs, and behaviours related to SCD. Future research should explore these aspects to provide a more comprehensive understanding of the factors influencing SCD awareness and management. Despite these limitations, the study's findings offer valuable insights for informing targeted interventions aimed at improving awareness and knowledge of SCD within the community.

## Conclusion

The study found out that majority of the participants 84.68% had low knowledge levels hence highlighting the need for targeted educational interventions to address the low knowledge gaps, particularly focusing on SCD screening. Additionally, schools were the primary source of information (69.87%), followed by family and friends (43.38%) and social media (35.58%) hence indicating that schools need to be equipped with all the necessary SCD related information as majority of the young people get to learn more about SCD from schools. Finally, as knowledge of SCD was associated with being of a Muslim religion and having ever heard of someone with SCD, interventions to improve SCD awareness, knowledge and screening could utilize religious institutions to pass on SCD related information.
